# Supplementary material for: Consumer Awareness, Perceptions and Avoidance of Ultra-Processed Foods: A Study of UK Adults in 2024
Source: Foods. 2024 Jul 23;13(15):2317. doi: 10.3390/foods13152317 (PMC11311829; doi:10.3390/foods13152317)
Supplement: Supplementary file 1 [file foods-13-02317-s001.zip › foods-3115705-supplementary.pdf]

<u>Supplementary Materials for: Consumer awareness, perceptions and avoidance of ultra-processed foods: A study of UK adults in 2024</u>

Out of the total of 10 UPFs and non-UPFs, 1% of participants categorised all correctly and no participants incorrectly categorised all 10. The median number of correct categori-sations was 6/10 (24% of participants). Of the 5 UPFs, 13% of participants categorised all 5 as UPF and 6% identified 0 as UPF. The median number of UPFs correctly categorised was 2/5 (21% of participants). Of the 5 non-UPFs, 25% correctly categorised all 5 as non-UPF and 4% identified 0 as non-UPF. The median number of non-UPFs correctly categorised was 4 (36%).

Sociodemographic predictors of 'do you know what UPFs are?' (yes vs. no/unsure)

White (vs. non-white) participants (OR = 1.53, $p < .001$), older age (OR=1.48, $p = .001$), higher education level (OR = 2.10, $p < .001$), quintile 5 (vs. quintile 1) household income (OR = 2.04, $p < .001$) were significantly more likely to report knowing what UPFs are. No other socio-demographic predictors were significant at $p < .01$.

Sociodemographic predictors of 'would you feel confident in identifying whether a food is UPF or not?' (yes vs. no/unsure)

Participants who were of older (≥40yrs) age (OR = 1.44, $p < .001$), had a higher edu-cation level (OR = 1.48, $p < .001$), had quintile 3 (vs. quintile 1) household income (OR = 1.53, $p = .002$) or quintile 5 (OR = 1.77, $p < .001$) were significantly more likely to report be-ing confident in identifying whether a food is UPF. No other socio-demographic predic-tors were significant at $p < .01$.

Accuracy for UPF identification (totals out of 5 items)

Female participants (B = .21, $p < .001$), older adults (B = .19, $p = .002$) participants with higher education levels (B = .49, $p < .001$) and the highest quintile (5 vs. 1) for household income (B = .41, $p < .001$) categorised more of the 5 UPFs accurately, on average.

Accuracy for non-UPF identification (totals out of 5 items)

Female participants (B = .-.19, $p < .001$), older adults (B = -.25, $p < .001$) participants with higher education levels (B = -.17, $p = .002$) and white participants (B = .23, $p = .004$) categorised fewer 5 non-UPFs accurately, on average.
